# The Controlling Nutritional Status Score as a Predictive Factor for Lung Metastasis in Patients with Hepatocellular Carcinoma After Hepatectomy

**DOI:** 10.3390/cancers18152523

**Published:** 2026-08-06

**Authors:** Jiro Kimura, Kosei Takagi, Tomokazu Fuji, Kazuya Yasui, Takeyoshi Nishiyama, Toshiyoshi Fujiwara

**Affiliations:** Department of Gastroenterological Surgery, Graduate School of Medicine, Dentistry, and Pharmaceutical Sciences, Okayama University, Okayama 700-8530, Japanpri958hs@s.okayama-u.ac.jp (T.F.); pjyv6nvp@s.okayama-u.ac.jp (K.Y.); me17063@s.okayama-u.ac.jp (T.N.); toshi_f@md.okayama-u.ac.jp (T.F.)

**Keywords:** hepatectomy, hepatocellular carcinoma, lung metastasis, Controlling Nutritional Status, predictive factor

## Abstract

**Background/Objectives:** Lung metastasis (LM) is the most common type of extrahepatic metastasis after hepatectomy for hepatocellular carcinoma. However, the predictive factors for LM after hepatectomy for hepatocellular carcinoma remain incompletely characterized. This study aimed to examine the predictive factors for LM after hepatectomy for hepatocellular carcinoma and clarify the association between preoperative biomarkers and LM. **Methods:** We analyzed data of 644 consecutive patients with hepatocellular carcinoma who underwent primary hepatectomy between July 2003 and December 2023. Patients were divided into two groups: LM (+) and LM (−). Additionally, the association between perioperative factors and LM was investigated. Subsequently, a risk model was developed to predict LM. **Results:** Of the 644 patients, 43 (6.7%) experienced LM. Regarding biochemical scores, only the proportion of high Controlling Nutritional Status (CONUT) score (≥3) was significantly different (43.9% in the LM (−) group vs. 60.5% in the LM (+) group, *p* = 0.04). In multivariable analysis, a high CONUT score, tumor size, and microvascular invasion were identified as independent predictive factors for LM. The risk model exhibited accuracy with an area under the curve of 0.93, 0.85, and 0.83 in the 1-, 3-, and 5-year LM, respectively. **Conclusions:** The present study demonstrated an association between CONUT score and LM after primary hepatectomy for hepatocellular carcinoma and found that a high CONUT score is a complementary predictive factor for LM.

## Simple Summary

Although hepatectomy is a potentially curative treatment for hepatocellular carcinoma, it is associated with a high rate of postoperative recurrence and metastasis. Lung metastasis is the most common type of extrahepatic metastasis and is a prognostic factor after hepatectomy. This study aimed to examine the predictive factors for lung metastasis after hepatectomy for hepatocellular carcinoma. We analyzed data of 644 consecutive patients who underwent primary hepatectomy. Among them, 43 (6.7%) experienced lung metastasis. In this study, a high Controlling Nutritional Status (CONUT) score, tumor size, and microvascular invasion were identified as independent predictive factors for lung metastasis. In conclusion, a high CONUT score is identified as a complementary predictive factor for lung metastasis after hepatectomy for hepatocellular carcinoma.

## 1. Introduction

Hepatocellular carcinoma (HCC) is a common form of liver cancer in many countries with an increasing incidence [1] and is the third leading cause of cancer-related **mortality** worldwide [2]. Current treatment for HCC is recommended using multimodal and high-intensity strategies such as local therapy, including surgical resection, ablation, and intra-arterial therapy, together with immunotherapy-based systemic therapy [3]. Although hepatectomy is a potentially curative treatment for HCC, it is associated with a high rate of postoperative recurrence and metastasis [4].

Lung metastasis (LM) is the most common type of extrahepatic metastasis, possibly because the circulatory pressure in the lungs is lower than that in other body areas, which may lead to the accumulation of liver cancer cells and occurrence of lung metastasis [5]. The treatment of LM remains controversial, with a 5-year overall survival (OS) rate of only 2.5% for synchronous HCC with LM [6]. Therefore, LM is a poor prognostic factor in patients with HCC.

Recent studies have suggested that inflammation is closely associated with tumorigenesis, cancer progression, and metastasis [7,8]. In addition, immunonutritional biochemical scores are used to predict the prognosis of other cancers and are also closely associated with the prognosis of HCC [9,10]. Various biochemical scores, including the Controlling Nutritional Status (CONUT) score, prognostic nutritional index (PNI), aspartate aminotransferase (AST)-to-platelet ratio index (APRI), platelet-to-lymphocyte ratio (PLR), and neutrophil-to-lymphocyte ratio (NLR), have been developed and are generally accepted by clinicians as predictors for OS and recurrence-free survival (RFS) after hepatectomy for HCC [11,12,13,14,15]. However, few studies have focused on the association between biochemical scores and postoperative LM [16]. Since we previously reported that the CONUT score is a valuable preoperative predictor of survival in patients undergoing hepatectomy for HCC, its significance for prognosis after hepatectomy has been clarified [11,12]. However, the association between the CONUT score and LM remains unclear, despite microvascular invasion (MVI) and tumor size being discussed as risk factors [17,18].

Although LM may occur as part of a broader metastatic phenotype, its relatively frequent occurrence and clinical relevance motivated us to examine LM as a clinically relevant endpoint. This study aimed to identify clinicopathological factors associated with LM after hepatectomy and to evaluate the association between preoperative biochemical scores, such as the CONUT score, and LM.

## 2. Materials and Methods

### 2.1. Patients and Study Design

This retrospective cohort study used a database of 823 consecutive patients with HCC who underwent hepatectomy at our institution (a tertiary care hospital) between July 2003 and December 2023. Patients undergoing repeat hepatectomy, those with extrahepatic metastases at the time of hepatectomy, and those with combined hepatocellular cholangiocarcinoma were excluded because of different recurrence and survival risks. The study was approved by the Ethics Committee of our institution (approval no. 2506-035) and was conducted in accordance with the Declaration of Helsinki. Owing to the retrospective nature of this study, the requirement for informed consent was waived.

### 2.2. Clinical Data

The demographic and clinical data extracted from our database included age, sex, body mass index, American Society of Anesthesiologists Physical Status, comorbidities, preoperative laboratory tests, liver function according to the Child–Pugh classification, hepatitis virus infection, and tumor markers. The biological scores were calculated using the following formulas: Albumin-Bilirubin (ALBI) score = [log10 bilirubin (μmol/L) × 0.66] + [albumin (g/L) × (−0.085)] [19]; APRI = (AST (IU/L)/upper limit of normal AST (IU/L)) × 100/platelet count (109/L) [20]; Fib-4 index = age (years) × AST (IU/L)/(platelet count (×109/L) × √(alanine aminotransferase (ALT) (IU/L))) [21]; PNI = (10 × albumin (g/dL)) + (0.005 × total lymphocyte count (/mm^3^)) [22]; and Model for End-Stage Liver Disease 3.0 score = 1.33 × (female) + 4.56 × ln(bilirubin) + 0.82 × (137 − sodium) − 0.24 × (137 − sodium) × ln(bilirubin) + 9.09 × ln(INR) + 11.14 × ln(creatinine) + 1.85 × (3.5 − albumin) − 1.83 × (3.5 − albumin) × ln(creatinine) + 6 [23]. The CONUT score was calculated as the sum of the individual scores for albumin level, total lymphocyte count, and total cholesterol level, as shown in Supplemental Table S1 [24]. In this study, a high CONUT score was defined as a CONUT score of ≥3, while the low CONUT score was defined as a CONUT score of <3. The cutoff value of CONUT score ≥3 was prespecified based on results of previous studies and was selected before the analysis of the present cohort [11]. Operative factors included hepatectomy, blood transfusion, extrahepatic bile duct resection, and resection of other organs. Pathological factors included TNM stage according to the Liver Cancer Study Group of Japan [25], size and number of tumors (solitary or multiple), lymph node metastases, MVI, differentiation, growth pattern, fibrosis, capsule formation, intrahepatic metastasis, and surgical margins. MVI was defined as the presence of microscopic tumor cell nests or emboli within the vascular spaces adjacent to the primary tumor, identified on histopathological examination. The presence or absence of MVI was retrospectively determined based on the original pathological reports of the resected specimens.

Postoperative surveillance was performed according to a standardized institutional protocol throughout the study period (2003–2023). The patients underwent contrast-enhanced chest and abdominal computed tomography (CT) every 3 months during the first 2 years after hepatectomy and every 6 months thereafter. The surveillance strategy remained consistent throughout the study period with no substantial modifications. LM was diagnosed based on radiological findings on serial chest CT imaging. When imaging findings were equivocal or when differentiation from primary lung cancer, or other pulmonary diseases was difficult, the diagnosis was established through a multidisciplinary evaluation by experienced thoracic radiologists and pulmonologists. LM was defined as the development of pulmonary metastasis at any time during follow-up, regardless of whether it was the first site of recurrence or occurred after recurrence at other sites. Long-term postoperative outcomes included the location of metastasis and the status of LM. The patients were divided into two groups according to their LM status.

### 2.3. Objective

The primary aim of this study was to investigate the association between preoperative biochemical scores and LM development after hepatectomy for HCC. As a secondary objective, we performed an exploratory analysis to assess whether these clinicopathological factors could be combined into a multivariable risk stratification model for estimating the cause-specific risk of LM at 1, 3, and 5 years after hepatectomy. The predictive analyses were intended to provide preliminary evidence for risk stratification rather than to establish a definitive clinical prediction model.

### 2.4. Statistical Analysis

Patient characteristics, surgical factors, and pathological factors stratified by the presence or absence of LM were evaluated. The cumulative incidence of LM was estimated using the cumulative incidence function, with death without LM treated as a competing event, using the cuminc() function in the cmprsk package in R (version 4.3.1; R Foundation for Statistical Computing, Vienna, Austria). Numbers of patients at risk at 1, 3, and 5 years were calculated based on the observed follow-up time. Cox proportional hazards regression analysis was performed to identify factors associated with the cause-specific hazard of LM after hepatectomy. For univariable analyses, patients with missing data for a given variable were excluded. A complete-case analysis was performed for the multivariable analysis, and patients with missing data for any variable included in the model were excluded. Variables with clinical relevance or statistical significance in the univariable analysis were included in the multivariable analysis.

Multivariable Cox proportional hazards regression analysis was performed to identify independent factors associated with LM after hepatectomy. The cause-specific risks of LM were estimated using the baseline survival function from the Cox model and each patient’s linear predictors. The proportional hazards assumption for the final Cox regression model was formally assessed using Schoenfeld residuals for each predictor and for the model. The time-dependent area under the curve (AUC) for the receiver operating characteristic (ROC) curve was calculated at 12, 36, and 60 months using censoring-adjusted time-dependent ROC analysis. Internal validation was performed using 1000 bootstrap re-samples [26]. The final three-predictor Cox model was refitted in each bootstrap sample, and the model performance was evaluated in both the bootstrap sample and the original dataset to estimate optimism. Predictor selection was not repeated within each bootstrap resample because the final predictor set was determined before internal validation. Calibration performance was evaluated by comparing the predicted and observed probabilities of LM.

Values are presented as proportions for categorical variables and as medians with interquartile ranges (IQRs) for continuous variables. Statistical comparisons were performed using the Mann–Whitney U test for continuous variables and Fisher’s exact test for categorical variables. All reported *p*-values were two-tailed, and a *p* value < 0.05 was considered statistically significant. All statistical analyses were performed using the EZR software (version 1.65; Saitama Medical Center, Jichi Medical University, Saitama, Japan).

## 3. Results

### 3.1. Study Cohort

**Figure 1:**
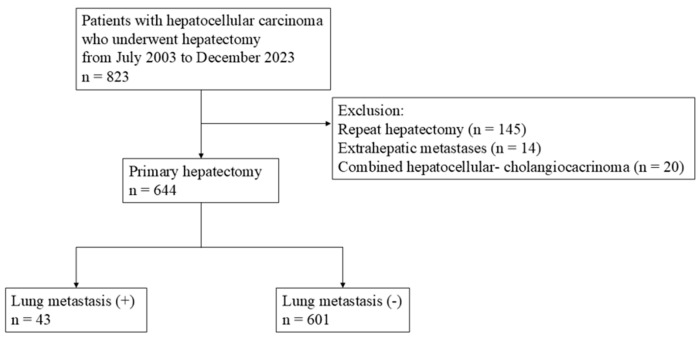
Flowchartof the study.

Of the 823 patients, data from 644 were analyzed after excluding 179 patients who underwent repeat hepatectomy (*n* = 145), extrahepatic metastases at the time of hepatectomy (*n* = 14), and combined hepatocellular cholangiocarcinoma (*n* = 20) (Figure 1). The characteristics of the 644 patients are summarized in Table 1. The cohort included 502 men (78.0%) and 142 women (22.0%) with a median age of 69.0 (IQR, 61.0–74.0) years. Bisectionectomy or trisectionectomy was performed in 213 (33.1%) patients, and the number of each pathological stage was 114 (17.7%), 320 (49.7%), 146 (22.7%), 64 (9.9%) for stages I, II, III, and IVa, respectively.

The perioperative characteristics stratified by LM status (LM (−) (*n* = 601) and LM (+) (*n* = 43)) are presented in Table 1. Alpha-fetoprotein (AFP) (*p* = 0.001) and protein levels induced by vitamin K absence or antagonist-II (PIVKA-II) (*p* < 0.001) were significantly higher in the LM (+) group. Among the evaluated biochemical scores, high CONUT score showed the strongest association with LM (43.9% in the LM (−) group vs. 60.5% in the LM (+) group, *p* = 0.04). Among the operative factors, no significant differences were observed between the groups except for the surgical procedure. Significant differences were observed in pathological stage (*p* < 0.001), maximum tumor size (*p* < 0.001), and MVI (*p* < 0.001).

### 3.2. The Site of Recurrence Stratified by the Status of LM Following Hepatectomy

**Figure 2:**
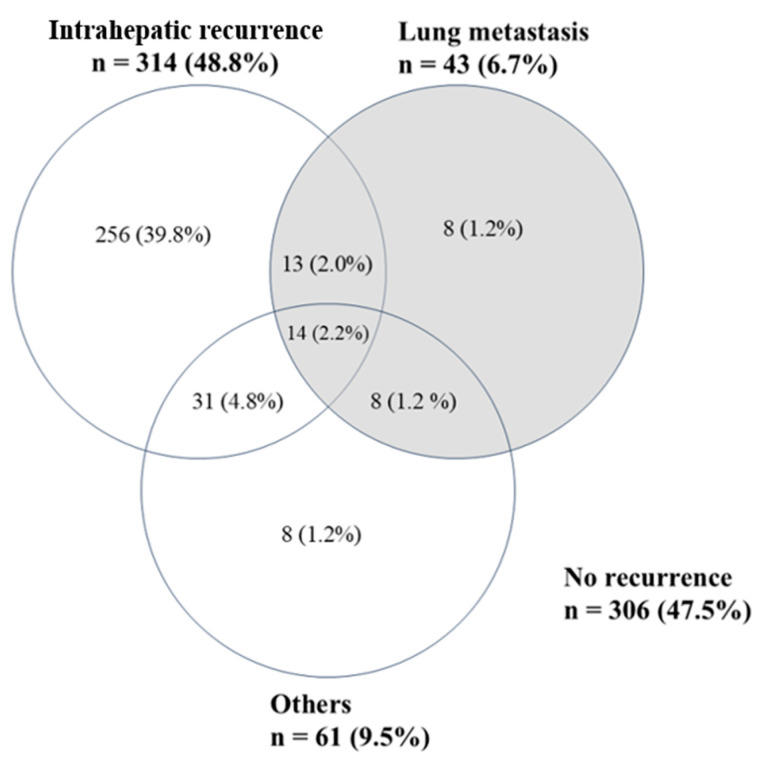
Relationship between recurrent sites of the tumor and the number of patients after hepatectomy.

After a median follow-up of 54.0 months, 217 (33.7%) died from the following causes: cancer progression (*n* = 131), infection (*n* = 17), other cancer progression (*n* = 11), liver failure (*n* = 9), or other causes (*n* = 49). During follow-up, 191 patients died without developing LM. In addition, 338 patients (52.5%) experienced tumor recurrence after hepatectomy. The relationship between tumor recurrence sites and the number of patients during postoperative surveillance after hepatectomy is shown in Figure 2. Of the 644 patients, 338 (52.5%) experienced recurrence after hepatectomy. Of these, 314 patients (48.8%) had intrahepatic recurrence and 43 (6.7%) had LM. Among the 43 patients with LM, 35 (81.4%) had other metastatic sites. Among patients who developed LM, the median time from hepatectomy to LM was 8.0 (4.0–16.5) months. The cumulative incidence of LM was 4.26% (95% CI, 2.65–5.86%) at 1 year, 6.43% (95% CI, 4.44–8.41%) at 3 years, and 6.87% (95% CI, 4.80–8.93%) at 5 years. The numbers of patients at risk at 1, 3, and 5 years were 526, 400, and 297, respectively (Supplemental Table S2).

Supplemental Table S3 shows the site of recurrence stratified by LM status. Between the two groups, the rate of intrahepatic recurrence was not significantly different (47.8% in LM (−) vs. 62.8% in LM (+), *p* = 0.06). The rates of lymph node metastasis (*p* < 0.001), peritoneal metastasis (*p* = 0.004), and bone metastasis (*p* < 0.001) were significantly higher in the LM (+) group. In addition, brain metastases were observed only in the LM (+) group (*p* < 0.001).

### 3.3. The Factors Associated with LM Following Hepatectomy

The results of the Cox proportional hazards analysis are presented in Table 2. In univariable analyses, high CONUT score (HR 1.93; 95% CI 1.04–3.56; *p* = 0.03), AFP ≥ 400 ng/mL (HR 2.52; 95% CI 1.23–5.13; *p* = 0.01), PIVKA-II ≥ 40 mAU/mL (HR 4.03; 95% CI 1.86–8.70; *p* < 0.001), type of hepatectomy (HR 2.53; 95% CI 1.39–4.61; *p* = 0.002), tumor size ≥ 5 cm (HR 6.45; 95% CI 3.40–12.22; *p* < 0.001) and MVI (HR 9.44; 95% CI 4.74–18.77; *p* < 0.001) were significantly associated with LM. Among the evaluated biochemical and immunonutritional scores, the CONUT score was significantly associated with the time to LM in the univariable Cox regression analysis. Among these variables, multivariable analyses showed a significant difference only in high CONUT score (HR 1.99; 95% CI 1.06–3.73; *p* = 0.03), tumor size ≥ 5 cm (HR 2.76; 95% CI 1.30–5.89; *p* = 0.008) and MVI (HR 5.24; 95% CI 2.33–11.81; *p* < 0.001).

### 3.4. Exploratory Risk Stratification Analysis for LM Following Hepatectomy

An exploratory risk stratification analysis for LM was performed based on the results of multivariable Cox proportional hazards regression analysis. In the final three-predictor model using CONUT, tumor size, and MVI, CONUT included 644 patients, of whom 43 patients developed LM. It showed a borderline association with LM (HR 1.83; 95% CI 0.99–3.37; *p* = 0.05), whereas tumor size and MVI remained significantly associated with LM (Table 3). The proportional hazards assumption was assessed using Schoenfeld residuals. No evidence of violation was observed for CONUT score (*p* = 0.73); however, significant departures from the proportional hazards assumption were observed for tumor size (*p* < 0.001) and MVI (*p* = 0.002), with a significant global test for the overall model (*p* < 0.001).

Given these findings, the Cox model-based risk estimates should be interpreted cautiously, as the proportional hazards assumption was not fully satisfied. Therefore, this analysis was considered exploratory and was performed to illustrate differences in LM risk according to clinicopathological characteristics rather than to establish a definitive prediction tool. The prognostic score and estimated cause-specific risk calculations are provided in the Supplemental Methods.

### 3.5. Exploratory Evaluation of Model Performance

The exploratory performance of the risk stratification model based on the ROC curve was shown in Supplemental Figure S1. The AUCs of the risk stratification model for LM at 1, 3, and 5 years were 0.93 (95% CI 0.90–0.96), 0.85 (95% CI 0.78–0.93), and 0.83 (95% CI 0.75–0.92), respectively. The apparent Harrell’s concordance index (C-index) was 0.828. Internal validation using 1000 bootstrap resamples yielded a mean optimism of 0.0035 and an optimism-corrected C-index of 0.825. Calibration plots demonstrated overall agreement between the predicted and censoring-adjusted observed probabilities of LM at 12, 36, and 60 months, although some deviation from the ideal 45-degree line was observed, particularly at higher predicted probabilities (Supplemental Figure S2). The corresponding Brier scores were 0.035, 0.051, and 0.054, respectively. These analyses were considered exploratory and were performed to assess the potential utility of the identified risk factors for risk stratification.

## 4. Discussion

The present study analyzed the association between biochemical scores, including the CONUT score, and LM in 644 patients after primary hepatectomy for HCC. Although we previously reported that a high CONUT score was significantly associated with poor OS and RFS (11, 12), few studies have reported an association between the CONUT score and LM in patients with HCC. The proportion of patients with high CONUT score was significantly higher in the LM group. Tumor size, MVI, and CONUT score were associated with the subsequent development of LM after hepatectomy. However, because most patients with LM also developed extrapulmonary recurrence, these factors may partly reflect aggressive tumor biology rather than a tendency to metastasize to the lungs.

In this study, 35 of 43 patients (81.4%) with LM also developed metastases at other sites after hepatectomy, including the lymph nodes and brain (Figure 2 and Supplemental Table S3), highlighting the aggressive metastatic behavior of these tumors. Therefore, identifying factors associated with the development of LM after hepatectomy may be clinically valuable for postoperative surveillance and risk stratification. Our multivariable analysis revealed that high CONUT score, tumor size, and MVI were factors associated with LM. Among these factors, the CONUT score and tumor size can be measured before surgery. In addition, the factors consisting of the CONUT score (albumin level, total lymphocyte count, and total cholesterol level) are routinely measured before surgery. Although the CONUT score was independently associated with the development of LM in the multivariable analysis, its effect size was more modest than those of established tumor-related factors, such as tumor size, and MVI. In addition, the present study did not formally quantify the incremental predictive value of the CONUT score beyond established tumor-related factors using measures such as changes in the AUC, net reclassification improvement, or decision curve analysis. Therefore, CONUT score should be interpreted as a potential complementary marker of LM risk rather than as a superior or independent determinant of LM risk. The clinical utility of incorporating the CONUT score into existing risk stratification models requires further validation in larger independent cohorts.

Although a variety of biochemical scores were examined in this study, only the CONUT score was significantly different between the LM (+) and the LM (−) groups (Table 1). To date, only a few studies have investigated the predictive factors for LM. Pathologically, incomplete capsule formation, MVI, number of tumors, and tumor size have been identified as predictive factors for LM [16,18]. However, in terms of biochemical scores, the CONUT score was not considered, although the MELD score, platelet-albumin-bilirubin score, NLR, lymphocyte-to-monocyte ratio (LMR), PLR, and PNI were not identified as predictive factors for LM [16,27].

The CONUT score is a screening tool designed to assess the nutritional status of the patients [24]. Serum albumin level, total lymphocyte count, and total cholesterol level are used to calculate the score (Supplemental Table S1). Serum albumin levels reflect systemic inflammation. Proinflammatory cytokines such as interleukin-6 and tumor necrosis factor-alpha contribute to the modulation of albumin synthesis in the liver, resulting in decreased serum albumin concentration [28]. Because cytokine-mediated inflammatory responses can accelerate cancer cell growth, these cytokines also play an important role in cancer proliferation and metastasis [29]. Another study identified a negative correlation between albumin and urokinase plasminogen activator surface receptor expression, suggesting a mechanism by which albumin suppresses HCC metastasis in human specimens [30]. The total lymphocyte count is also an important biomarker of immune status, as lymphocytes are strongly associated with host cancer-mediated immunity to inhibit tumor proliferation and migration [31,32]. T lymphocyte apoptosis in rat models has been reported to positively correlate with the number of lung metastatic nodules in HCC [33]. Another study of HCC in a mouse model reported that lymphocyte infiltration reduced the number of microvessels around the tumor [34]. Serum total cholesterol level is a biomarker of nutritional status [35]. In addition, it is associated with tumor growth and prognosis in patients with various types of tumors, including HCC [36,37]. The combination of these three factors in the CONUT score can enhance its ability to accurately assess immuno-nutritional status. To date, the association between LM of HCC and these factors still lacks evidence, as mentioned above, and requires further research.

The present study has certain limitations. Although the sample size of 644 patients was relatively large, this was a single-center retrospective study conducted at a high-volume Japanese center, which may have contributed to a selection bias. Owing to the retrospective nature of the study, some data on clinical parameters were missing. In addition, treatment history before hepatectomy and the intent of hepatectomy were not clarified in the present study. To minimize bias, we excluded patients who underwent repeat hepatectomies and those with concomitant hepatocellular cholangiocarcinoma. Furthermore, the study period spanned two decades (2003–2023), during which substantial changes occurred in imaging modalities, surgical techniques, perioperative management, and systemic therapies for HCC. These temporal changes may have influenced LM detection, recurrence patterns, and survival outcomes. In this study, the Cox proportional hazards model was used to identify factors associated with LM. Exploratory analyses were additionally performed to assess the potential utility of these factors for risk stratification. As death before the occurrence of LM represents a competing event, the Cox model does not directly estimate the absolute cumulative incidence of LM. Furthermore, the proportional hazards assumption was not fully satisfied in the final Cox model, as violations were observed for tumor size and MVI. Therefore, the hazard ratios for these variables may not remain constant over time, and the exploratory risk stratification analyses should be interpreted with caution. Because predictor selection was not repeated within each bootstrap sample, the optimism correction may not fully capture optimism related to variable selection. Finally, the current analyses were only validated in an internal validation cohort. Therefore, this model should be externally validated in future studies. Future studies using statistical approaches that accommodate time-varying effects and external validation cohorts are warranted to further evaluate the generalizability and clinical applicability of these findings.

## 5. Conclusions

The present study demonstrated an association between CONUT score and LM after primary hepatectomy for HCC. A high CONUT score may provide additional prognostic information when considered together with established clinicopathological factors. Further multicenter studies are warranted to validate these findings and clarify the clinical utility of CONUT-based risk stratification.

## Figures and Tables

**Table 1:** Patient characteristics according to the status of lung metastasis.

Variables		Total (*n* = 644)	LM (−) (*n* = 601)	LM (+) (*n* = 43)	*p* Value
*Patient characteristics*					
Age, years		69.0 [61.0, 74.0]	69.0 [61.0, 74.0]	67.0 [61.0, 73.0]	0.57
Sex	Male	502 (78.0)	466 (77.5)	36 (83.7)	0.45
	Female	142 (22.0)	135 (22.5)	7 (16.3)	
BMI, kg/m^2^		23.3 [21.0, 25.6]	23.4 [21.1, 25.7]	21.9 [20.5, 23.9]	0.02
ASA-PS score	1–2	549 (85.2)	510 (84.9)	39 (90.7)	0.38
	3–4	95 (14.8)	91 (15.1)	4 (9.3)	
Comorbidity	CH/LC	406 (63.0)	384 (63.9)	22 (51.2)	0.10
	COPD	19 (3.0)	19 (3.2)	0 (0.0)	0.63
	Hypertension	129 (20.0)	121 (20.1)	8 (18.6)	1.00
	Chronic heart failure	19 (3.0)	18 (3.0)	1 (2.3)	1.00
	CVD	16 (2.5)	14 (2.3)	2 (4.7)	0.29
	CKD	18 (2.8)	18 (3.0)	0 (0.0)	0.63
	Diabetes	159 (24.7)	149 (24.8)	10 (23.3)	1.00
Albumin, g/dL		3.9 [3.6, 4.2]	4.0 [3.7, 4.2]	3.8 [3.5, 4.2]	0.15
Total bilirubin, mg/dL		0.72 [0.55, 0.99]	0.72 [0.56, 0.99]	0.75 [0.53, 1.01]	0.89
Platelet, ×10^4^/μL		16.1 [12.0, 21.5]	16.0 [12.0, 21.3]	20.2 [15.7, 28.7]	0.002
Child–Pugh grade	A	625 (97.0)	582 (96.8)	43 (100.0)	0.63
	B	19 (3.0)	19 (3.2)	0 (0.0)	
HBsAg		133 (20.7)	125 (20.8)	8 (18.6)	0.85
HCVAb		170 (26.4)	165 (27.5)	5 (11.6)	0.02
AFP, ng/mL		8.1 [3.5, 59.7]	7.3 [3.5, 45.3]	64.5 [5.0, 361.4]	0.001
PIVKA-II, mAU/mL		52.5 [22.0, 662.5]	48.5 [22.0, 476.2]	516.5 [50.0, 23,793.0]	<0.001
ALBI score		−2.62 [−2.88, −2.35]	−2.62 [−2.89, −2.36]	−2.51 [−2.84, −2.33]	0.19
APRI		0.69 [0.45, 1.17]	0.69 [0.44, 1.17]	0.78 [0.50, 1.18]	0.68
CONUT score	≥3	290 (45.0)	264 (43.9)	26 (60.5)	0.04
FIB-4 index		2.67 [1.80, 4.09]	2.69 [1.80, 4.09]	2.65 [1.80, 3.94]	0.77
PNI		45.9 [42.3, 49.9]	46.1 [42.6, 50.0]	43.9 [41.0, 48.4]	0.09
MELD 3.0		11.5 [10.6, 12.4]	11.5 [10.5, 12.4]	11.5 [10.7, 12.2]	0.75
*Surgical factors*					
Type of hepatectomy	bisectionectomy or more	213 (33.1)	190 (31.6)	23 (53.5)	0.004
Blood transfusion		151 (23.4)	136 (22.6)	15 (34.9)	0.09
BDR		5 (0.8)	4 (0.7)	1 (2.3)	0.29
Other organ resection		46 (7.1)	42 (7.0)	4 (9.3)	0.54
*Pathological factors*					
Pathological stage	I	114 (17.7)	112 (18.6)	2 (4.7)	<0.001
	II	320 (49.7)	311 (51.7)	9 (20.9)	
	III	146 (22.7)	126 (21.0)	20 (46.5)	
	IVa	64 (9.9)	52 (8.7)	12 (27.9)	
Tumor size, cm	≥5	185 (28.7)	156 (26.0)	29 (67.4)	<0.001
Tumor number	≥2	125 (19.4)	113 (18.8)	12 (27.9)	0.16
Lymph node metastases		7 (1.1)	6 (1.0)	1 (2.3)	0.39
MVI		189 (29.3)	157 (26.1)	32 (74.4)	<0.001
Poor differentiation		70/615 (11.4)	62/573 (10.8)	8/42 (19.0)	0.13
Infiltrative growth		36 (5.6)	33 (5.5)	3 (7.0)	0.73
Fibrosis grade	≥3	289/638 (45.3)	275/595 (46.2)	14/43 (32.6)	0.11
Capsule formation		367 (57.0)	338 (56.2)	29 (67.4)	0.20
Positive Surgical margin		17 (2.6)	15 (2.5)	2 (4.7)	0.32

Values are reported as median [IQR] or number (percentage). AFP, alpha-fetoprotein; ALBI, albumin-bilirubin index; APRI, aspartate aminotransferase to platelet ratio index; ASA-PS, American Society of Anesthesiologists Physical Status; BDR, bile duct resection; BMI, body mass index; CH, chronic hepatitis; CKD, chronic kidney disease; CONUT, Controlling Nutritional Status; COPD, chronic obstructive pulmonary disease; CVD, cerebrovascular disease; FIB-4, Fibrosis-4; HBsAg, hepatitis B antigen; HCVAb, hepatitis C antibody; LC, liver cirrhosis; LM, lung metastasis; MELD, Model for End-Stage Liver Disease; MVI, microvascular invasion; PIVKA-II, protein induced by vitamin K absence-II; PNI, prognostic nutritional index.

**Table 2:** Univariate and multivariate analyses of factors associated with lung metastasis in patients who underwent hepatectomy.

			Univariate			Multivariate		
Variables		No. of Patients	HR	95% CI	*p* Value	HR	95% CI	*p* Value
Age; years	≥65	406	1.10	0.59–2.03	0.77			
Sex	Male	502	1.47	0.65–3.29	0.36			
BMI; kg/m^2^	≥25	200	0.51	0.23–1.10	0.09			
Platelet; ×10^4^/μL	≤10	96	0.71	0.28–1.81	0.48			
Serum albumin; g/dL		-	0.64	0.33–1.22	0.17			
Total lymphocyte count (/mm^3^)		-	0.99	0.99–1.00	0.24			
Total cholesterol (mg/dL)		-	1.01	0.99–1.02	0.06			
CONUT score	≥ 3	290	1.93	1.04–3.56	0.03	1.99	1.06–3.73	0.03
ALBI	>−2.60	307	1.77	0.96–3.27	0.07			
APRI	>1.5	115	0.61	0.24–1.56	0.31			
FIB-4 index	≥2.67	322	0.84	0.46–1.53	0.57			
PNI	≤40	91	1.40	0.62–3.15	0.42			
MELD 3.0	≥14	79	0.86	0.31–2.41	0.77			
AFP; ng/mL	≥400	84	2.52	1.23–5.13	0.01	1.21	0.57–2.53	0.61
PIVKA-II; mAU/mL	≥40	343	4.03	1.86–8.70	<0.001	1.50	0.64–3.51	0.36
Type of hepatectomy	Bisectione-ctomy or more	213	2.53	1.39–4.61	0.002	0.75	0.38–1.49	0.41
Tumor size; cm	≥5	185	6.45	3.40–12.22	<0.001	2.76	1.30–5.89	0.008
Tumor number	≥2	125	1.65	0.84–3.20	0.14			
Lymph node metastases		7	3.14	0.43–22.85	0.26			
MVI		189	9.44	4.74–18.77	<0.001	5.24	2.33–11.81	< 0.001
Infiltrative growth		36	1.42	0.44–4.60	0.56			
Capsule formation		367	1.56	0.82–2.95	0.17			
Positive Surgical margin		17	2.27	0.54–9.40	0.26			

AFP, alpha-fetoprotein; ALBI, albumin-bilirubin index; APRI, aspartate aminotransferase to platelet ratio index; BMI, body mass index; CI, confidence interval; CONUT, Controlling Nutritional Status; FIB-4, Fibrosis-4; HR, hazard ratio; MELD, Model for End-Stage Liver Disease; MVI, microvascular invasion; PIVKA-II, protein induced by vitamin K absence-II; PNI, prognostic nutritional index.

**Table 3:** Final three-predictor Cox regression model for lung metastasis.

			Multivariate			
Variables		No. of Patients	β Coefficient	HR	95% CI	*p* Value
CONUT score	≥3	290	0.5935	1.83	0.99–3.37	0.05
Tumor size; cm	≥5	185	1.0532	2.88	1.42–5.88	0.004
MVI		189	1.7391	5.69	2.65–12.24	<0.001

CI, confidence interval; CONUT, Controlling Nutritional Status; HR, hazard ratio; MVI, microvascular invasion.

## Data Availability

All data supporting the findings of this study are available within the paper and its Supplementary Materials.

## Supplementary Materials

The following supporting information can be downloaded at: https://www.mdpi.com/article/10.3390/cancers18152523/s1, Table S1: Details of CONUT score; Table S2: Cumulative incidence of lung metastasis after hepatectomy for hepatocellular carcinoma; Table S3: The site of recurrence stratified by the status of lung metastasis; Figure S1: Receiver operating characteristic curves of the Cox proportional hazards-based risk model for lung metastasis after hepatectomy. The model performance for predicting lung metastasis at 1 year (a), 3 years (b), and 5 years (c) after hepatectomy is shown; Figure S2: Calibration plot of the risk stratification model for lung metastasis at 1 year (a), 3 years (b) and 5 years (c). The plots show the relationship between predicted and censoring-adjusted observed probabilities of lung metastasis. The diagonal line represents perfect calibration.

## Abbreviations

LM, Lung metastasis; CONUT, Controlling Nutritional Status; HCC, hepatocellular carcinoma; OS, overall survival; PNI, prognostic nutritional index; AST, aspartate aminotransferase; APRI, aspartate aminotransferase-to-platelet ratio; PLR, platelet-to-lymphocyte ratio; NLR, neutrophil-to lymphocyte ratio; RFS, recurrence-free survival; MVI, microvascular invasion; BMI, body mass index; ASA-PS, American Society of Anesthesiologists Physical Status; ALBI, Albumin-Bilirubin; ALT, alanine aminotransferase; MELD, Model for End-Stage Liver Disease; INR, international normalized ratio; CT, computed tomography; HR, hazard ratio; CI, confidence interval; AUC, area under the curve; ROC, receiver operating characteristic; IQR, interquartile range; AFP, alpha-fetoprotein; PIVKA-II, protein induced by vitamin K absence or antagonist-II.

## References

[1] Yang J.D., Hainaut P., Gores G.J., Amadou A., Plymoth A., Roberts L.R. (2019). A global view of hepatocellular carcinoma: Trends, risk, prevention and management. Nat. Rev. Gastroenterol. Hepatol..

[2] Bray F., Ferlay J., Soerjomataram I., Siegel R.L., Torre L.A., Jemal A. (2018). Global cancer statistics 2018: GLOBOCAN estimates of incidence and mortality worldwide for 36 cancers in 185 countries. CA Cancer J. Clin..

[3] Xie D., Shi J., Zhou J., Fan J., Gao Q. (2023). Clinical practice guidelines and real-life practice in hepatocellular carcinoma: A Chinese perspective. Clin. Mol. Hepatol..

[4] Niu Z.S., Wang W.H., Niu X.J. (2022). Recent progress in molecular mechanisms of postoperative recurrence and metastasis of hepatocellular carcinoma. World J. Gastroenterol..

[5] An X., Li F., Mou C., Li D. (2021). A systematic review and meta-analysis on prognosis and survival of hepatocellular carcinoma with lung metastasis after hepatectomy. Ann. Palliat. Med..

[6] Hu Z., Huang P., Zhou Z., Li W., Xu J., Xu K., Wang J., Zhang H. (2018). Aggressive intrahepatic therapies for synchronous hepatocellular carcinoma with pulmonary metastasis. Clin. Transl. Oncol..

[7] Aguilar-Cazares D., Chavez-Dominguez R., Marroquin-Mucino M., Perez-Medina M., Benito-Lopez J.J., Camarena A., Rumbo-Nava U., Lopez-Gonzalez J.S. (2022). The systemic-level repercussions of cancer-associated inflammation mediators produced in the tumor microenvironment. Front. Endocrinol..

[8] Khandia R., Munjal A. (2020). Interplay between inflammation and cancer. Adv. Protein Chem. Struct. Biol..

[9] Goktas Aydin S., Cakan Demirel B., Bilici A., Topcu A., Aykan M.B., Kahraman S., Akbiyik I., Atci M.M., Olmez O.F., Yaren A. (2022). Real-life analysis of treatment approaches and the role of inflammatory markers on survival in patients with advanced biliary tract cancer. Curr. Med. Res. Opin..

[10] Sanghera C., Teh J.J., Pinato D.J. (2019). The systemic inflammatory response as a source of biomarkers and therapeutic targets in hepatocellular carcinoma. Liver Int..

[11] Takagi K., Yagi T., Umeda Y., Shinoura S., Yoshida R., Nobuoka D., Kuise T., Araki H., Fujiwara T. (2017). Preoperative Controlling Nutritional Status (CONUT) Score for Assessment of Prognosis Following Hepatectomy for Hepatocellular Carcinoma. World J. Surg..

[12] Takagi K., Domagala P., Polak W.G., Buettner S., Ijzermans J.N.M. (2019). Prognostic significance of the controlling nutritional status (CONUT) score in patients undergoing hepatectomy for hepatocellular carcinoma: A systematic review and meta-analysis. BMC Gastroenterol..

[13] Ji F., Liang Y., Fu S.J., Guo Z.Y., Shu M., Shen S.L., Li S.Q., Peng B.G., Liang L.J., Hua Y.P. (2016). A novel and accurate predictor of survival for patients with hepatocellular carcinoma after surgical resection: The neutrophil to lymphocyte ratio (NLR) combined with the aspartate aminotransferase/platelet count ratio index (APRI). BMC Cancer.

[14] Mao S., Yu X., Sun J., Yang Y., Shan Y., Sun J., Mugaanyi J., Fan R., Wu S., Lu C. (2022). Development of nomogram models of inflammatory markers based on clinical database to predict prognosis for hepatocellular carcinoma after surgical resection. BMC Cancer.

[15] Wu Y., Tu C., Shao C. (2021). Inflammatory indexes in preoperative blood routine to predict early recurrence of hepatocellular carcinoma after curative hepatectomy. BMC Surg..

[16] Zhou H., Zheng H., Wang Y., Lao M., Shu H., Huang M., Ou C. (2024). Nomogram for Predicting Postoperative Pulmonary Metastasis in Hepatocellular Carcinoma Based on Inflammatory Markers. Cancer Control.

[17] Ishii T., Hatano E., Yasuchika K., Taura K., Seo S., Uemoto S. (2014). High risk of lung metastasis after resection of hepatocellular carcinoma more than 7 cm in diameter. Surg. Today.

[18] Li J., Liu Y., Yan Z., Wan X., Xia Y., Wang K., Liu J., Lau W.Y., Wu M., Shen F. (2014). A nomogram predicting pulmonary metastasis of hepatocellular carcinoma following partial hepatectomy. Br. J. Cancer.

[19] Johnson P.J., Berhane S., Kagebayashi C., Satomura S., Teng M., Reeves H.L., O’Beirne J., Fox R., Skowronska A., Palmer D. (2015). Assessment of liver function in patients with hepatocellular carcinoma: A new evidence-based approach-the ALBI grade. J. Clin. Oncol..

[20] Wai C.T., Greenson J.K., Fontana R.J., Kalbfleisch J.D., Marrero J.A., Conjeevaram H.S., Lok A.S. (2003). A simple noninvasive index can predict both significant fibrosis and cirrhosis in patients with chronic hepatitis C. Hepatology.

[21] Shah A.G., Lydecker A., Murray K., Tetri B.N., Contos M.J., Sanyal A.J., Nash Clinical Research Network (2009). Comparison of noninvasive markers of fibrosis in patients with nonalcoholic fatty liver disease. Clin. Gastroenterol. Hepatol..

[22] Onodera T., Goseki N., Kosaki G. (1984). [Prognostic nutritional index in gastrointestinal surgery of malnourished cancer patients]. Nihon Geka Gakkai Zasshi.

[23] Kim W.R., Mannalithara A., Heimbach J.K., Kamath P.S., Asrani S.K., Biggins S.W., Wood N.L., Gentry S.E., Kwong A.J. (2021). MELD 3.0: The Model for End-Stage Liver Disease Updated for the Modern Era. Gastroenterology.

[24] Ignacio de Ulibarri J., Gonzalez-Madrono A., de Villar N.G., Gonzalez P., Gonzalez B., Mancha A., Rodriguez F., Fernandez G. (2005). CONUT: A tool for controlling nutritional status. First validation in a hospital population. Nutr. Hosp..

[25] Kudo M., Kitano M., Sakurai T., Nishida N. (2015). General Rules for the Clinical and Pathological Study of Primary Liver Cancer, Nationwide Follow-Up Survey and Clinical Practice Guidelines: The Outstanding Achievements of the Liver Cancer Study Group of Japan. Dig. Dis..

[26] Eforn B. (1979). Bootstrap methods: Another look at the jackknife. Ann. Stat..

[27] Huang W.J., Wang G.Y., Liu Z.Y., Zhang M.L., Wang W., Zhang X., Wang R.T. (2022). Preoperative PDW levels predict pulmonary metastasis in patients with hepatocellular carcinoma. BMC Cancer.

[28] Peters S.J., Vanhaecke T., Papeleu P., Rogiers V., Haagsman H.P., van Norren K. (2010). Co-culture of primary rat hepatocytes with rat liver epithelial cells enhances interleukin-6-induced acute-phase protein response. Cell Tissue Res..

[29] Mantovani A., Allavena P., Sica A., Balkwill F. (2008). Cancer-related inflammation. Nature.

[30] Fu X., Yang Y., Zhang D. (2022). Molecular mechanism of albumin in suppressing invasion and metastasis of hepatocellular carcinoma. Liver Int..

[31] Gajewski T.F., Schreiber H., Fu Y.X. (2013). Innate and adaptive immune cells in the tumor microenvironment. Nat. Immunol..

[32] Kaida T., Nitta H., Kitano Y., Yamamura K., Arima K., Higashi T., Taki K., Nakagawa S., Okabe H., Hayashi H. (2017). Preoperative platelet-to-lymphocyte ratio can predict recurrence beyond the Milan criteria after hepatectomy for patients with hepatocellular carcinoma. Hepatol. Res..

[33] Xia Y.H., Wang Z.M., Chen R.X., Ye S.L., Sun R.X., Xue Q., Huang Y. (2013). T-cell apoptosis induced by intratumoral activated hepatic stellate cells is associated with lung metastasis in hepatocellular carcinoma. Oncol. Rep..

[34] Yamashita Y.I., Shimada M., Hasegawa H., Minagawa R., Rikimaru T., Hamatsu T., Tanaka S., Shirabe K., Miyazaki J.I., Sugimachi K. (2001). Electroporation-mediated interleukin-12 gene therapy for hepatocellular carcinoma in the mice model. Cancer Res..

[35] de Ulíbarri Pérez J.I., Fernández G., Rodríguez Salvanés F., Díaz López A.M. (2014). Nutritional screening; control of clinical undernutrition with analytical parameters. Nutr. Hosp..

[36] Kang H.W., Seo S.P., Kim W.T., Yun S.J., Lee S.C., Kim W.J., Hwang E.C., Kang S.H., Hong S.H., Chung J. (2018). Low preoperative serum cholesterol level is associated with aggressive pathologic features and poor cancer-specific survival in patients with surgically treated renal cell carcinoma. Int. J. Clin. Oncol..

[37] Lee Y.L., Li W.C., Tsai T.H., Chiang H.Y., Ting C.T. (2016). Body mass index and cholesterol level predict surgical outcome in patients with hepatocellular carcinoma in Taiwan—A cohort study. Oncotarget.

