# Supplementary material for: The Controlling Nutritional Status Score as a Predictive Factor for Lung Metastasis in Patients with Hepatocellular Carcinoma After Hepatectomy
Source: Cancers (Basel). 2026 Aug 6;18(15):2523. doi: 10.3390/cancers18152523 (PMC13465628; doi:10.3390/cancers18152523)
Supplement: Supplementary file 1 [file cancers-18-02523-s001.zip › cancers-4447073-supplementary.pdf]

Supplemental Table S1 Details of CONUT score

	Classification of undernutrition			
	Normal	Mild	Moderate	Severe
Serum albumin (g/dL)	≥ 3.5	3.0 - 3.49	2.5 - 2.99	< 2.5
Score	0	2	4	6
Total lymphocyte count (/mm³)	≥ 1600	1200 - 1599	800 - 1199	< 800
Score	0	1	2	3
Total cholesterol (mg/dL)	≥ 180	140 - 179	100 - 139	< 100
Score	0	1	2	3
Total score	0 - 1	2 - 4	5 - 8	9 - 12

CONUT, controlling nutritional status.

Supplemental Table S2 Cumulative incidence of lung metastasis after hepatectomy for hepatocellular carcinoma.

Time	Number at risk	LM events	Death without LM	LM cumulative incidence, %	95% CI
1 year	526	26	30	4.26	2.65–5.86
3 years	400	38	83	6.43	4.44–8.41
5 years	297	40	117	6.87	4.80–8.93

LM, lung metastasis

Supplemental Table S3 The site of recurrence stratified by the status of lung metastasis

	Total (n = 644)	LM (-) (n = 601)	LM (+) (n = 43)	P value
Liver	314 (48.8)	287 (47.8)	27 (62.8)	0.06
Lung	43 (6.7)	0 (0)	43 (100)	< 0.001
Lymph node	19 (3.0)	11 (1.8)	8 (18.6)	< 0.001
Peritoneum	11 (1.7)	7 (1.2)	4 (9.3)	0.004
Bone	24 (3.7)	16 (2.7)	8 (18.6)	< 0.001
Brain	4 (0.6)	0 (0)	4 (9.3)	< 0.001
Adrenal gland	4 (0.6)	3 (0.5)	1 (2.3)	0.24
Tumor thrombus	5 (0.8)	4 (0.7)	1 (2.3)	0.29
Stomach	1 (0.2)	1 (0.2)	0 (0)	1.00
Spleen	1 (0.2)	1 (0.2)	0 (0)	1.00
Muscle	1 (0.2)	1 (0.2)	0 (0)	1.00
Skin	1 (0.2)	0 (0)	1 (2.3)	0.07

Values are reported as number (percentage).

Supplemental Figure Legends

Supplemental Figure S1. Receiver operating characteristic curves of the Cox proportional hazards-based risk model for lung metastasis after hepatectomy. The model performance for predicting lung metastasis at 1 year (a), 3 years (b), and 5 years (c) after hepatectomy is shown.

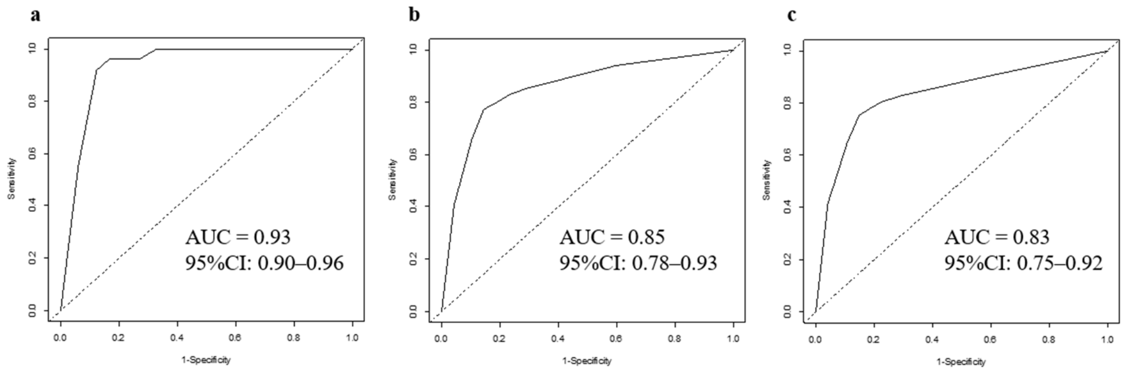

Supplemental Figure S2. Calibration plot of the risk stratification model for lung metastasis at 1 year (a), 3 years (b) and 5 years (c). The plots show the relationship between predicted and censoring-adjusted observed probabilities of lung metastasis. The diagonal line represents perfect calibration.

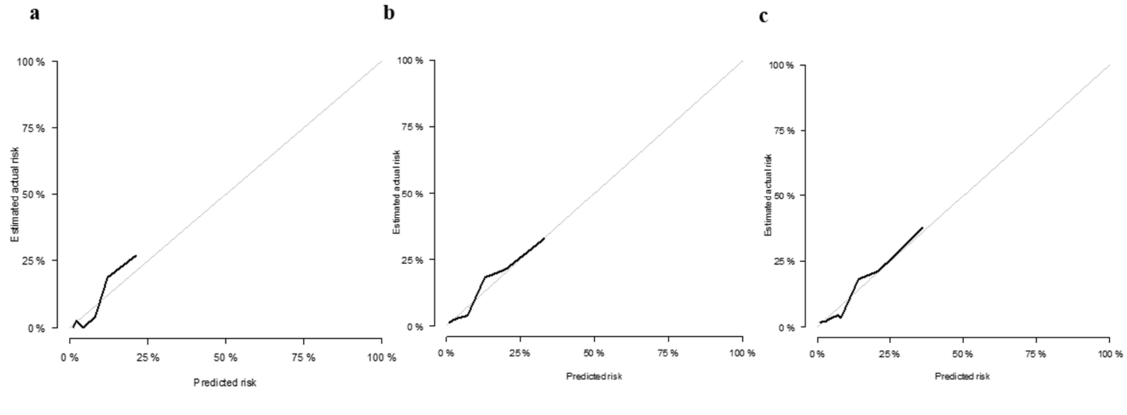

Supplemental Methods

Exploratory risk score calculation

An exploratory risk score was calculated based on the coefficients derived from the multivariable Cox proportional hazards regression model including CONUT score, tumor size, and MVI.

The LP was calculated as follows:

$LP=0.5935 \times (CONUT \geq 3) + 1.0532 \times (Tumor\ size \geq 5\ cm) + 1.7391 \times (MVI)$

The estimated cause-specific risk of LM at time months after hepatectomy was calculated using:

$P(t)\ (\%) = 100 – 100 * \{S_0(t) \wedge exp(LP)\}$

$= 100 − 100 * exp[-H_0(t) * exp\{0.5935 * (CONUT\ score \geq 3)$

$+ 1.0532 * (Tumor\ size \geq 5\ cm) + 1.7391 * (MVI)\}]$

The baseline cumulative hazards used for exploratory estimation were:

$H_0(12) = 0.008004$, $H_0(36) = 0.013342$, $H_0(60) = 0.014695$

Definitions:

LP, linear predictor

P, estimated cause-specific risk of LM

t, Time (months) after hepatectomy

$S_0(t)$, baseline survival function

$H_0(t)$, baseline cumulative hazard
